# Human parainfluenza 2 & 4: Clinical and genetic epidemiology in the UK, 2013–2017, reveals distinct disease features and co‐circulating genomic subtypes

**DOI:** 10.1111/irv.13012

**Published:** 2022-06-07

**Authors:** Akhil Chellapuri, Matthew Smitheman, Joseph G. Chappell, Gemma Clark, Hannah C. Howson‐Wells, Louise Berry, Jonathan K. Ball, William L. Irving, Alexander W. Tarr, C. Patrick McClure

**Affiliations:** ^1^ School of Life Sciences University of Nottingham Nottingham UK; ^2^ Wolfson Centre for Global Virus Research University of Nottingham Nottingham UK; ^3^ Clinical Microbiology Nottingham University Hospitals NHS Trust Nottingham UK

**Keywords:** Human parainfluenza 2, Human parainfluenza 4, Orthorubulavirus, Human orthorubulavirus 2, Human orthorubulavirus 4, respiratory infection

## Abstract

**Background:**

Human Parainfluenza viruses (HPIV) comprise of four members of the genetically distinct genera of Respirovirus (HPIV1&3) and Orthorubulavirus (HPIV2&4), causing significant upper and lower respiratory tract infections worldwide, particularly in children. However, despite frequent molecular diagnosis, they are frequently considered collectively or with HPIV4 overlooked entirely. We therefore investigated clinical and viral epidemiological distinctions of the relatively less prevalent Orthorubulaviruses HPIV2&4 at a regional UK hospital across four autumn/winter epidemic seasons.

**Methods:**

A retrospective audit of clinical features of all HPIV2 or HPIV4 RT‐PCR‐positive patients, diagnosed between 1st September 2013 and 12th April 2017 was undertaken, alongside sequencing of viral genome fragments in a representative subset of samples.

**Results:**

Infection was observed across all age groups, but predominantly in children under nine and adults over 40, with almost twice as many HPIV4 as HPIV2 cases. Fever, abnormal haematology, elevated C‐reactive protein and hospital admission were more frequently seen in HPIV2 than HPIV4 infection. Each of the four seasonal peaks of either HPIV2, HPIV4 or both, closely matched that of RSV, occurring in November and December and preceding that of Influenza A. A subset of viruses were partially sequenced, indicating co‐circulation of multiple subtypes of both HPIV2&4, but with little variation between each epidemic season or from limited global reference sequences.

**Conclusions:**

Despite being closest known genetic relatives, our data indicates a potential difference in associated disease between HPIV2 and HPIV4, with more hospitalisation seen in HPIV2 mono‐infected individuals, but a greater overall number of HPIV4 cases.

## 1 INTRODUCTION

Human parainfluenza viruses types 1 to 4 (HPIV1–4) are collectively the second most common cause of hospitalisation for children under the age of five, behind only Respiratory Syncytial Virus (RSV).^1^, ^2^, ^3^, ^4^ Symptomatic HPIV infection is observed in both adults and children worldwide, affecting both the upper and lower respiratory tract^5^, ^6^ with varying severity in the immunocompromised and elderly.^7^, ^8^ HPIV2 presents generally with common cold‐like symptoms and is a frequent cause of croup in infants.^2^, ^9^ HPIV4 is less well characterised but has been associated with bronchiolitis and pneumonia.^5^, ^10^ Parainfluenza virus infections place a significant burden on the global healthcare system. In the US alone, a 12‐year retrospective study estimated hospital charges for children under the age of five annually totalled in excess of $42 and $57 million for HPIV‐associated bronchiolitis and pneumonia, respectively, with a gross yearly HPIV associated US hospitalisation burden estimated at 62000 days.^11^

HPIVs belong to the single‐stranded negative sense RNA *Paramyxoviridae* family and are sub‐divided to the *Respirovirus* genus (HPIV1&3) of the *Orthoparamyxovirinae* subfamily and the significantly genetically distinct *Orthorubulavirus* genus (HPIV2&4) of the *Rubulavirinae* subfamily.^12^ HPIV2&4 present an orthodox six gene Paramyxovirus genome,^12^ with particularly the Hemagglutinin‐Neuraminidase (HN) and also Fusion (F) envelope genes known to display a higher level of antigenic variation than the structural components of the orthorubulavirus genome, making them a more appropriate focus for epidemiological studies.^13^, ^14^, ^15^, ^16^

Two nearly identical archetypal strains of HPIV2 have been described: Greer in the mid‐1950s in the US^17^ and Toshiba in the late 1970s in Japan.^18^ More recently, additional lineage defining strains Vanderbilt 94 and 98 have been characterised, with maximal dissimilarity rates circa 5% at the amino acid level.^13^ In contrast HPIV4 is categorised into two different subtypes (HPIV4A and HPIV4B) based on antigenic properties,^19^, ^20^ despite presenting apparently less divergent genomes^16^ which do not meet criteria for demarcation as separate species.^12^ To date no distinction in clinical outcome has been made amongst circulating HPIV2 or 4 species, further illustrated by phylogenetic studies indicating genetically related clades often contain strains from different seasons and distant geographical origins.^15^, ^16^

Formative viral diagnostic protocols reliant on cell culture (commonly with primary rhesus monkey kidney cells) resulted in low recovery rates, little cytopathic effect and a weak haemadsorption pattern.^9^, ^19^, ^21^ This has in part led to HPIV4's frequent omission from standard diagnostic respiratory investigation and derivation of minimal reference genome data, in turn contributing to a reduced comprehension of its epidemiological significance.^9^, ^10^, ^15^, ^22^ Higher sensitivity and specificity of reverse transcription PCR (RT‐PCR)^23^ and its cost‐effective ability to rapidly diagnose viral respiratory infection has improved HPIV surveillance in the current millennium. Recent advances in sequencing technologies should further redress this shortfall in genomic reference material.^24^

To further increase our knowledge of HPIV2&4 clinical epidemiology, and the differences between the two infections, we undertook a retrospective analysis of all RT‐PCR‐positive samples at a regional UK diagnostic laboratory between September 2013 and April 2017. We additionally sequenced a sub‐sample of archived genomic extracts to characterise the underlying complementary genetic epidemiology occurring in this study period.

## 2 METHODS

### 2.1 Samples and routine diagnostic assessment

Clinical specimens were obtained for routine diagnosis from sputum, nasopharyngeal aspirate or throat swab samples from patients with suspected respiratory virus infections in primary and secondary care units in the Nottingham University Hospitals Trust catchment area between 1st September 2013 and 12th April 2017. Nucleic acids were extracted and screened by an in house respiratory virus diagnostic panel and stored as previously described^25^ until March 2016 when a commercial screening panel was adopted (Ausdiagnostics), adding Coronavirus and Enterovirus detection.^26^ Available higher titre HPIV2&4‐positive nucleic acids with a cT value of lower than 33 (HPIV2) or 30 (HPIV4) as determined by in house RT‐qPCR or with a value greater than 100 (HPIV2) or 1000 (HPIV4) copies per 10 μL RNA, as determined by the AusDiagnostics assay were selected for further genetic investigation.

### 2.2 RT‐PCR, primer design and sequencing

cDNA was synthesized from archived nucleic acid extracts with RNA to cDNA EcoDry™ Premix containing random hexamers (Takara Clontech) as per the manufacturer's instruction. 1 μL of this RT reaction was used for PCR comprising of 1x HotStarTaq PCR buffer (QIAGEN), 3 pmol each primer, 400 μM total dNTPs, 0.375 U HotStarTaq DNA polymerase (QIAGEN) and molecular grade water in a 15 μL volume, then thermocycled at 95 °C for (15 min), followed by 55 cycles at 95 °C for 20 sec, annealing temperature (see supplementary Table S2) for 20 sec and 72 °C for 60 sec, then a final additional 72 °C for 2 min. cDNA integrity was initially assessed with a modified pan‐orthorubulavirus assay generating a 224 bp product using primers AVU‐RUB‐F2 & AVU‐RUB‐R.^27^ All available complete HPIV2&4 F & HN gene sequences were downloaded from GenBank in October 2017 (NCBI:txid 2 560 525 & 2 560 526 respectively) and used to design species‐specific primers targeting the envelope genes. Ultimately, three primer combinations were used to generate amplicons (and the resulting sequence data presented herein): HPIV2_Fs and HPIV2_Fas, HPIV2_Fs2 and HPIV2_Fas2, and HPIV4_Fs and HPIV4_FasINT (Supplementary Table S1). PCR products of expected size when examined by agarose gel electrophoresis were subjected to Sanger sequencing (Source BioScience, Nottingham, UK). Sequence identity was confirmed using NCBI Standard Nucleotide BLAST (BLASTn). Sequences derived were deposited in GenBank under accession numbers MZ576382:MZ576430.

### 2.3 Phylogenetic analysis

Sequences were analysed using MEGA6 software. Study sequences were aligned using ClustalW with reference sequences retrieved from NCBI databases in June 2021, by BLASTN searching for related sequences with complete coverage across the regions amplified, using HPIV2&4 reference genomes AF533010 & AB543336 as bait. All retrieved sequences were assigned to either NCBI:txid2560525 & 2 560 526 and matched with identity equal or greater than 91.75 or 87.45%, for HPIV2&4 bait regions respectively. A test of phylogenetic models indicated the Hasegawa‐Kishino‐Yano model with gamma distribution best fit and with invariant sites best fit for HPIV2 and HPIV4 data respectively. Maximum likelihood phylogenetic trees with 1000 bootstraps were constructed using the selected models.

### 2.4 Statistical analyses

All data analyses were performed using Microsoft Excel and GraphPad PRISM 7.04.

Comparison of clinical parameters was performed using Graphpad Prism (v9.3.1) statistical software. Similar to previous studies,^9^, ^22^ binary logistic regression comparing HPIV2 and HPIV4 infections was not conducted due to the occurrence of incomplete data for some individuals, limiting our ability to model the contributions of some parameters to a logistic regression. As such, categorical datasets were compared using either Fisher's exact test or χ2 tests. Median values of continuous datasets were compared using Mann–Whitney tests.

## 3 RESULTS

### 3.1 Routine diagnostic surveillance

Within the study period from 1st September 2013 to 11th April 2017, 26 593 unique respiratory specimens were investigated by routine RT‐PCR viral screening, of which 10 283 (38.15%) were positive for one or more viruses (data not shown). Of the positive specimens, 121 (1.18%) presented HPIV2 and 238 (2.31%) HPIV4. Additional positive specimens included 30.76% Rhino & enterovirus, 20.16% Respiratory Syncytial Virus, 12.19% Influenza A, 10.23% Adenovirus, 6.23% HPIV3, 5.55% Human Metapneumovirus, 5.65% Coronavirus (tested only from March 2016 onwards), 3.28% Influenza B and 2.75% HPIV1. Deduplication of multi‐sampled patients yielded 112 and 199 individuals infected with HPIV2 and HPIV4 respectively. Of these, 41.96% of HPIV2 and 43.72% of HPIV4 individuals were co‐infected with another pathogen.

### 3.2 Seasonality

**FIGURE 1:**
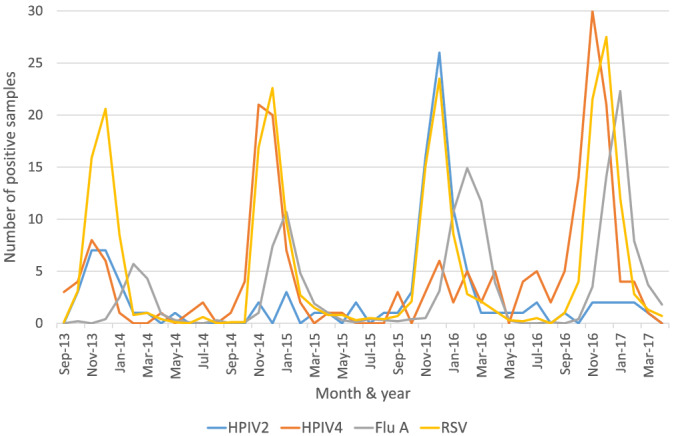
HPIV2&4 RT‐PCR‐positive samples compared to influenza A and RSV by month, between 1st September 2013 and 11th April 2017 in Nottingham UK. RSV and Influenza A numbers have been reduced tenfold to aid clarity

HPIV2 infections peaked around December in both 2013 and 2015, with approximately 4‐fold more cases in 2015 than 2013 (Figure 1). HPIV4 presented a more complex pattern, with significant spikes of cases around December of both 2014 and 2016, but also a smaller increase of positivity was observed in the autumn/winter of 2013, comparable in magnitude to the HPIV2 caseload (Figure 1). The epidemic Human orthorubulavirus season generally began in October and ended in January, peaking in November and December of each year, although sporadic cases of both HPIV2&4 were detected between the biennial peaks each year (Figure 1).

When compared with the most prevalent seasonal viral pathogens, RSV and Influenza A, each peak of orthorubulavirus cases closely matched those of RSV, although the surge in HPIV4 cases slightly preceded the RSV epidemic in the 2016/17 epidemic period. Both HPIV2&4 case spikes always preceded the Influenza A epidemic observed in January and February of each year (Figure 1). Human orthorubulavirus infection appeared independent of co‐presentation with RSV, with only 11.6% of HPIV2 and 14.57% of HPIV4 instances of co‐positivity (data not shown).

### 3.3 Demographics

**FIGURE 2:**
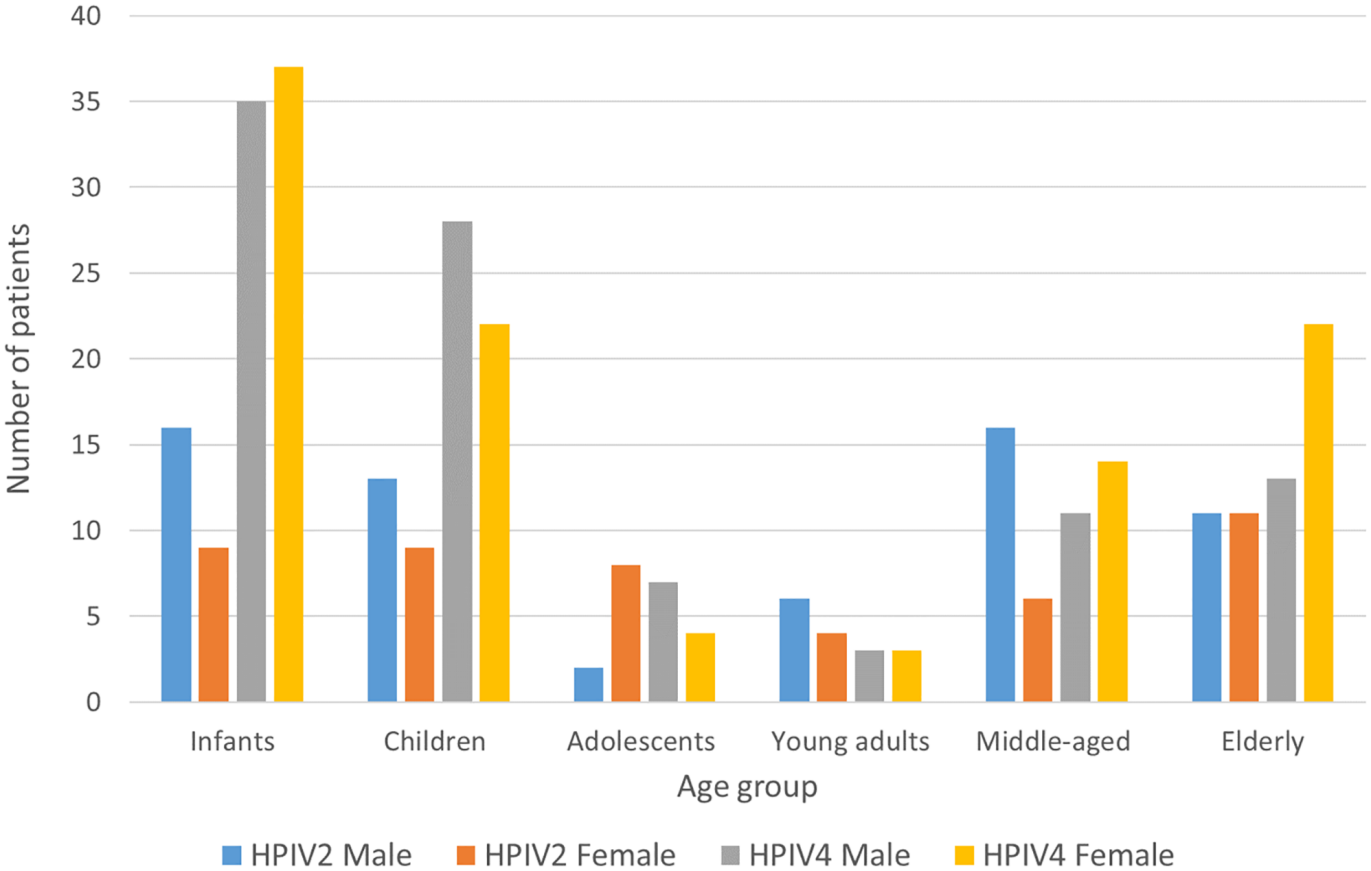
Age and sex distribution of HPIV2&4‐positive patients between 1st September 2013 and 11th April 2017. Age group categories are defined by new‐born to 1 year old (infants), 2 to 9 (children), 10 to 19 (adolescents), 20 to 40 (young adults), 41 to 64 (middle aged) and ≥ 65 (elderly)

Patients were categorised into gender and six age groups, then assessed for HPIV2 & 4 prevalence (Figure 2). For both types of orthorubulavirus, an increase in cases was observed towards the extremes of age, i.e. in infants/young children and older adults, with low numbers of infections observed in those aged 10 to 40 (adolescents and young adults). This effect was most pronounced in HPIV4 with 61.31% of cases in children under 10 years of age, in contrast to HPIV2 with just 42.86% (Figure 2 and Table 1). The 65 and over elderly group comprised almost one fifth of cases for both HPIV2 (19.64%) and HPIV4 (17.59%, Figure 2 and Table 1).

**TABLE 1:** Characteristics of hospitalised HPIV2&4 mono‐infected individuals

*Characteristic*	HPIV2	HPIV4	P value
	*No. (%)*	*No. (%)*	
**Hospitalised fraction of mono‐infected individuals**	**56 (86.1)**	**75 (67.0)**	n/a
**Female sex**	28 (50.0)	35 (46.7)	0.7269
**Age (median)**	21.5 years	4	0.0527
*0–9 years*	24 (42.9)	47 (62.7)	n/a
*10–64 years*	15 (26.8)	13 (17.3)	n/a
*65 + years*	17 (30.4)	16 (21.3)	n/a
**Duration of stay (median)**	5 days	5 days	0.8761
range (days)	1–47	1–77	n/a
**Underlying medical conditions** †	45 (81.82)	62 (84.93)	0.6393
**Immunocompromised** †	25 (44.64)	31 (64.58)	0.0501
**Symptoms**			
Shortness of breath †	17 (31.48)	32 (42.67)	0.2053
Cough †	15 (27.78)	20 (26.67)	>0.9999
Coryza †	10 (18.52)	6 (8)	0.1037
Wheeze †	1 (1.85)	6 (8)	0.2376
Fever †	26 (48.15)	11 (14.67)	**<0.0001**
Poor feeding †	3 (5.56)	6 (8)	0.7336
**Medical interventions**			
antibiotics	25 (48.08)	42 (57.53)	0.3636
nebuliser	5 (9.43)	9 (12.33)	0.7761
oxygen	5 (9.43)	9 (12.33)	0.7761

† *Where data available for variable; n/a = not applicable; p values <0.05 highlighted in bold*.

### 3.4 Genetic epidemiology

To investigate the underlying genotype of the HPIV2&4‐positive patients identified, additional RT‐PCR was performed on a subsample of higher titre surplus nucleic acid from diagnostic screening and subjected to Sanger sequencing. Available residual samples with higher viral template quantity (see methods) were retrieved and subjected to an array of priming combinations targeting the Fusion (F) and Hemagglutinin (HN) envelope genes, designed with available sequences deposited on GenBank circa September 2017 (see supplementary information).

Two HPIV2 primer pairs successfully generated overlapping amplicons (1517 bp coverage) for the majority of samples tested (20 of 25), allowing phylogenetic analysis of the near complete Fusion gene. HPIV4 amplification proved more challenging, but a single primer pair successfully generated amplicons and 793 bp of sequence from the Fusion gene for 29 of 33 samples attempted.

**FIGURE 3:**
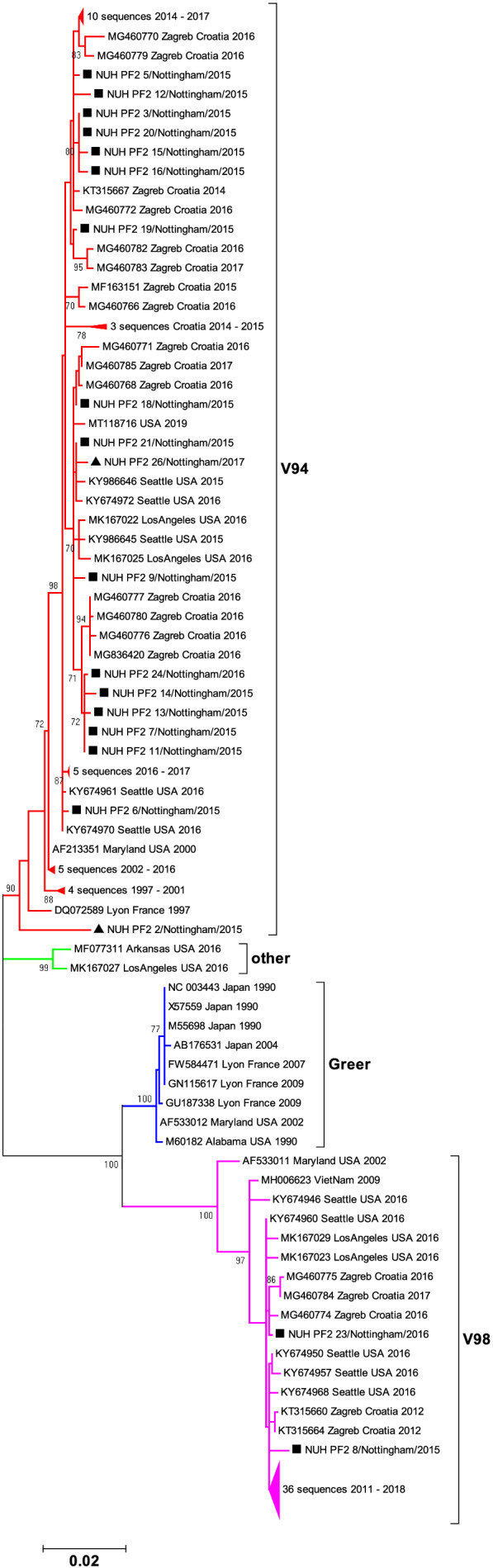
Phylogeny of HPIV2 study strains collected in Nottingham UK between 16th June 2015 and 26th January 2017 compared to available reference sequences derived from GenBank in June 2021 under the taxonomic designation NCBI:Txid 2 560 525. Sequences cover 1517 nucleotides from 4870 to 6386 of reference strain V94, GenBank accession no. AF533010. The unrooted circular tree was constructed by the maximum likelihood method with 1000 bootstraps; only bootstrap values with support greater than 70 are indicated alongside a scale bar showing genetic distance. Filled squares and triangles represent study samples collected during and outside the HPIV2 autumn/winter 2014/15 epidemic season respectively. Nominal V98‐like (pink), Greer‐like (blue), novel (green) and V94‐like (red) clade designations are annotated by subtree branch colour; selected subtrees have been collapsed for clarity, with sequence number and year range noted.

HPIV2 phylogeny in totality was suggestive of three well‐supported clades worldwide, defined by the three archetypal strains Greer, V94, V98,^13^ but unlike HPIV4 it is not formally designated into subtypes (Figure 3). Our recently sampled cohort indicated a predominance of sequences (n = 18 of 20 total) identifying as ‘V94‐like’ (red‐branched clade, Figure 3), with only two sequences ‘V98‐like’ (from patients aged four and 82, pink‐branched clade, Figure 3) and none clustering in either the ‘Greer‐like’ clade or an additional well‐supported clade comprised of two sequences from the USA in 2016 (blue and green‐branched clades respectively, Figure 3). Broad distribution across V‐94‐ and V98‐like clades was also observed for contemporary HPIV2 reference strains reported predominantly in Seattle, USA and Zagreb, Croatia. However, due to availability of residual samples with sufficient viral template, in addition to scarcity of reference HPIV2 strains in general, our data was biased toward the autumn/winter 2015/16 season. Only two isolates from outside this period were included (annotated by triangles in Figure 3). A January 2017 sequence was intermingled with majority of the V94‐like autumn/winter 2015/16 isolates, however an ‘out of season’ HPIV2 from June 2015 appeared genetically distinct from the majority of V94‐like isolates, suggesting a further unsampled diversity in HPIV2.

**FIGURE 4:**
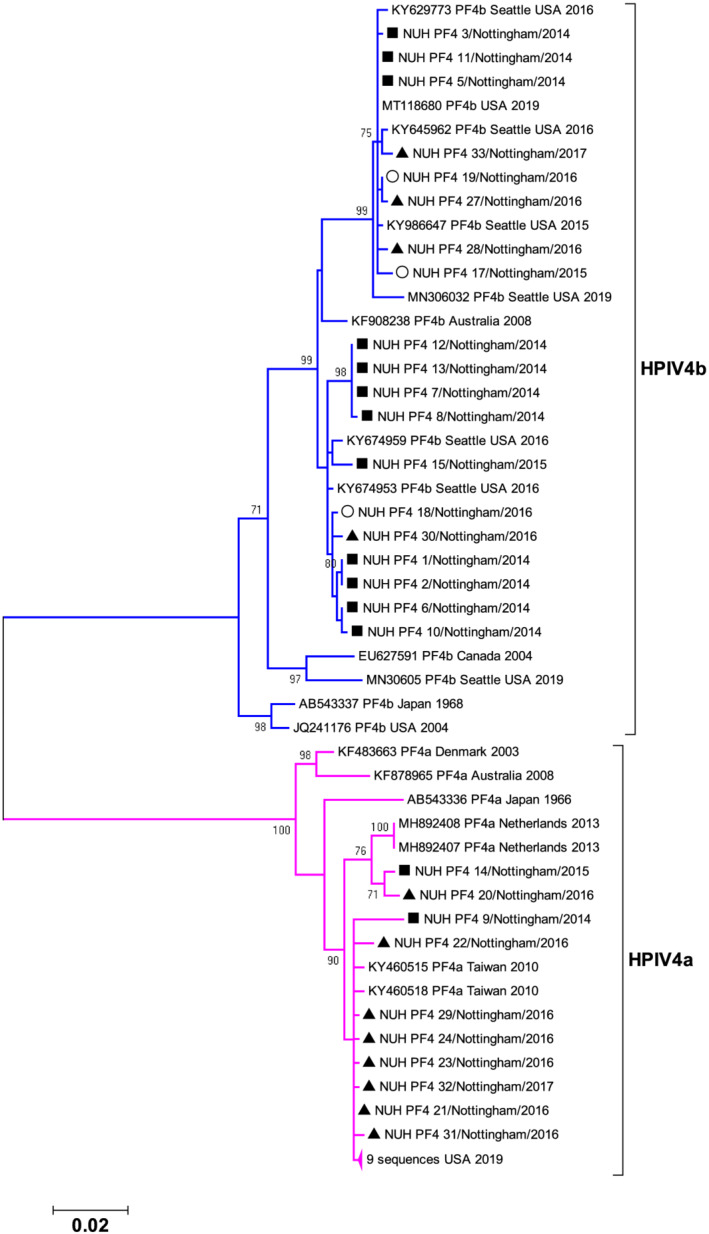
Phylogeny of HPIV4 study strains collected in Nottingham UK between 21st November 2014 and 23rd February 2017 compared to available reference sequences derived from GenBank in June 2021 under the taxonomic designation NCBI:Txid 2 560 526. Sequences cover 793 nucleotides from 5310 to 6107 of HPIV4A reference strain M‐25, GenBank accession no. AB543336. The unrooted circular tree was constructed by the maximum likelihood method with 1000 bootstraps; only bootstrap values with support greater than 70 are alongside a scale bar showing genetic distance. Filled squares and triangles represent study samples collected during the core HPIV4 2014/15 and 2016/17 autumn/winter epidemic seasons respectively, whilst open circle samples were collected in the autumn/winter of 2015/16. HPIV4A (pink) and HPIV4B (blue) subtype designations are annotated by subtree branch colour. 2014 study sequences NUH_PF4_1 & 2, and 12 & 13 represent paired samples from the same patient

Despite both a shorter analysed sequencing region and available reference genomes (28 only as of June 2021) relative to HPIV2, the HPIV4 phylogeny indicated a well‐supported division of designated subtypes HPIV4A&B (pink and blue‐branched clades respectively, Figure 4). Our sampled isolates from autumn/winter 2014/15 (Figure 4, filled squares) and 2016/17 (Figure 4, filled triangles) were represented and intermingled in both subtype HPIV4A and HPIV4B. Three ‘out of season’ (Figure 4, open circles) sequences from autumn/winter 2015/16 were exclusively 4b, but genetically indistinct from the 2014/15 and 2016/17 autumn/winter epidemic season samples. Both HPIV4A&B subtypes appear to further divided by two well‐supported distinct lineages, again all represented by sequences derived in different epidemic years (Figure 4).

### 3.5 Clinical characteristics

Significant incidence of both co‐infection and outpatient assessment was observed in the cohort, and HPIV infection could potentially be coincidental to a primary medical condition. Therefore, a focussed clinical audit was undertaken to compare features of hospitalised HPIV2 or HPIV4 mono‐infected patients (56 and 75 individuals respectively) where routine diagnostic investigation could reasonably rule out mild and co‐infection, to reduce confounding effects (Tables 1 & 2). However, in general, there were proportionally fewer hospital admissions for HPIV4‐positive individuals (p < 0.0001, data not shown).

**TABLE 2:** Investigations of hospitalised HPIV2&4 mono‐infected individuals

*Characteristic*	HPIV2	HPIV4	P value
	*No. (%)*	*No. (%)*	
**Radiological investigations**			
Chest X‐ray performed	28 (50)	43 (57.33)	0.4791
Radiological evidence of infection †	15 (55.56)	17 (38.64)	0.2205
**Haematological investigations**			
Blood tested	45 (80.36)	53 (70.67)	0.2284
	*low/normal/high*	*low/normal/high*	
*Haemoglobin level* †	26/18/1 (57.8/40.0/2.2)	17/34/2 (32.1/64.2/3.8)	**0.0146**
*Platelet count* †	14/27/4 (31.1/60/8.9)	9/41/3 (17.0/77.4/5.7)	0.309
*Red Blood cell count* †	24/20/1 (53.3/44.4/2.2)	4/49/0 (7.6/49/0)	**<0.0001**
*White Blood cell count* †	17/19/9 (37.8/42.2/20)	1/52/0 (1.9/98.1/0.0)	0.1293
*Neutrophil count* †	17/15/13 (37.8/33.3/28.9)	7/25/21 (13.2/47.2/39.6)	**0.0224**
*Lymphocyte count* †	25/19/1 (55.6/42.2/2.2)	25/27/1 (47.2/50.9/1.9)	0.461
*Eosinophil count* †	31/14/0 (68.9/31.1/0)	22/27/4 (41.5/50.9/7.55)	**0.0028**
*Basophil count* †	9/35/1 (20/77.8/2.2)	12/41/0 (22.6/77.4/0)	0.575
*Monocytic cell count* †	10/32/3 (22.2/71.1/6.7)	2/45/6 (3.8/84.9/11.3)	**0.0136**
**Liver and kidney assessment**			
Liver Function tested	37 (66.1)	33 (44.0)	**0.0139**
	*normal/high*	*normal/high*	
*AST level* †	32/2 (94.4/5.6)	31/1 (9.0/3.0)	>0.999
*ALT level* †	29/7 (80.6/19.4)	25/8 (75.8/24.2)	0.772
*Bilirubin level* †	33/3 (91.7/8.3)	27/6 (81.8/18.2)	0.294
*Albumin level* †	9/27 (25/75)	5/28 (15.2/84.9)	0.377
CRP tested	40 (71.4)	43 (57.3)	0.1039
	*normal/high*	*normal/high*	
*CRP level* †	14/26 (35.0/65.0)	28/15 (65.1/34.9)	**0.0084**
Kidney Function tested	44 (78.57)	42 (56)	**0.0091**
	*low/normal/high*	*low/normal/high*	
*Sodium level* †	8/35/1 (18.2/75.6/2.3)	11/28/3 (26.2/66.7/7.1)	0.765
*Potassium level* †	5/39/0 (11.4/88.5/0)	6/31/5 (14.3/73.8/11.9)	0.3279
*Urea level* †	11/26/7 (25.0/59.1/15.9)	11/22/9 (26.2/52.4/21.4)	0.7615
*Creatinine level* †	7/29/8 (15.9/65.9/18.2)	4/30/8 (9.5/71.4/19.1)	0.5464
**Cerebrospinal fluid tested**	2 (3.57)	7 (9.33)	0.2994

† *Where data available for variable. Abbreviations: AST Aspartate Aminotransferase, ALT Alanine Transaminase, CRP* C‐reactive protein*; p values <0.05 highlighted in bold*.

Although almost twice as many HPIV4 mono‐infected patients under the age of 10 were observed, the median ages were ultimately not statistically significant (p = 0.0527, Table 1). Most strikingly, a fever was observed with more than 3‐fold frequency in HPIV2 than HPIV4 patients (p < 0.0001, Table 1) but no further significant differences were seen in variables assessed. For both HPIV2 and HPIV4‐infected and hospitalised patients, underlying medical conditions were very common as was immunocompromise (Table 2). Other than the aforementioned fever associated with HPIV2, shortness of breath and cough were the most commonly recorded symptoms, but intervention with nebulisers and supplementary oxygen was only required in a minority of instances; antibiotics were provided to approximately half of the cohort (Table 2).

Patient blood was assessed more frequently in general than chest x‐rays, with significantly more haematological aberrance outside normal parameters (i.e. both low and high levels, Supplementary Table S2) seen in HPIV2 but not HPIV4 patients. Specifically, more frequently observed low haemoglobin levels (p = 0.0146) and low counts for red blood cells (p < 0.0001), neutrophils (p = 0.0224), eosinophils (p = 0.0028) and monocytes (p = 0.0136) were significant features of HPIV2 but not HPIV4 infection. Conversely, no differences were observed between HPIV2&4 for platelets, white blood cells, total lymphocytes and basophils (Table 2).

Liver and kidney function were assessed more frequently in HPIV2 patient care (p = 0.0139 and 0.0091 respectively, Table 2). However, of the many parameters tested, a significant disparity was seen only in the more frequently abnormally high C‐reactive protein levels of HPIV2 infection (p = 0.0084, Table 2). In general, abnormally high albumin but not bilirubin levels were recorded whilst urea levels appeared marginally more frequently outside standard ranges than sodium, potassium and creatinine (Table 2).

## 4 DISCUSSION

In contrast to more prevalent pre‐SARS‐CoV‐2 respiratory pathogens such as Influenza A and RSV, the Human Parainfluenza viruses are considerably under‐studied, yet still present a significant burden to global healthcare.^11^, ^24^ Previous key clinical studies have compared all four genetically distant types from different *Paramyxoviridae* sub‐families together,^9^, ^28^ excluded certain age groups^9^, ^22^ or overlooked HPIV4 entirely^22^ and lacked complementary genetic investigation. Even reports addressing genetic analyses have been limited in both patient and sequence numbers and clinical detail.^14^, ^15^, ^16^ The data presented here therefore represents to our knowledge the largest combined clinical and genetic study to date focussing exclusively on the epidemic human Orthorubulaviruses HPIV2&4.

Our retrospective observational period covered the principal epidemic autumn/winter period in 4 years, whereby we noted the biennial epidemic incidence previously attributed to HPIV2.^2^, ^3^, ^10^ A more complex pattern presented for HPIV4 was also consistent with other large‐scale studies^3^, ^5^, ^11^ alternating major and minor yearly epidemic seasons.

Many studies have previously observed a greater total incidence of diagnosed HPIV2 than HPIV4 cases,^28^ including significant nationwide studies in the US^10^ and the UK.^3^ Notably the UK study deriving data from 158 laboratories between 1998 and 2013 observed twice as many positive HPIV2 than HPIV4 tests.^3^ In contrast, we observed an almost twofold predominance of HPIV4 in general agreement with recent studies in China,^6^, ^29^ Vietnam^16^ and the USA.^9^, ^30^ This may be due to previous difficulty in culturing and related omission of HPIV4 from some diagnostic panels, increasing prevalence of HPIV4 or a combination of both factors.^3^, ^10^ Notably Zhao and colleagues^3^ detected a gradual increase of HPIV4 from 1998 through 2013, thus our contrasting results in the immediately subsequent period of 2013 to 2017 may actually indicate a further shift in the prevailing epidemiology of the human Orthorubulaviruses, in the UK at least. Continued uptake of HPIV4 in routine RT‐PCR surveillance and national‐level data aggregation^3^, ^10^ will further elucidate the true incidence and seasonality of HPIV4 and its prevalence relative to HPIV2.

Although greater sequence coverage allows more in‐depth resolution and interrogation of parainfluenza molecular epidemiology,^14^ even our relatively limited genetic investigation demonstrated contemporary co‐circulation of genetically distinct subtypes of both HPIV2 and HPIV4 in each of the four yearly epidemic seasons. This finding is consistent with previous investigation of HPIV2&4 in Vietnam^16^ and Croatia.^14^ Furthermore the predominance of Nottingham ‘V94‐like’ HPIV2 sequences was also seen in Vietnam and Croatia, where a shift from ‘G3’^14^ or ‘clade 1’^16^ sequences analogous to our ‘V98‐like’ designation between 2009 and 2014 to G1a/clade 2/V94‐like between 2014 and 2017 was observed.^31^ This apparent pattern of genotypic replacement may be driven by population level immunity and susceptibility,^31^ however the clade under‐represented by European sequences post 2014 was conversely over‐represented by an unpublished cohort of reference sequences from Seattle, USA (NCBI Bioproject PRJNA338014).

HPIV4 genetic epidemiology is much less understood, with a considerable sequence archive paucity relative to even HPIV2, to such an extent that we were able to compare our 29 Fusion gene sequences to only 28 publicly available references. Although somewhat paradoxically, and in contrast to HPIV2, the apparent clades of HPIV4 have been accepted as subtypes A and B in the literature. This may have arisen by chance through early isolation of distinct HPIV4 strains in contrast to highly similar early HPIV2 strains,^17^, ^18^, ^19^, ^20^ although HPIV4A&B are not considered to be distinct species.^12^ Our data indicates the apparent increase in HPIV4 incidence in the UK^3^ and possibly elsewhere involves multiple lineages of both HPIV4A&B, which we and others have demonstrated can cause clinical disease.^32^ Even this most populous genetic study of HPIV4 to date is however under‐powered to explore whether these subtypes and subtype lineages have different clinical properties, so like others^24^ we would urge for increased sequencing allied to future clinical studies, alongside population level serological investigation. Fluctuating and currently changing climate conditions may also have a current and future role in the clinical and genetic epidemiology of parainfluenza and other respiratory infections.^33^, ^34^ We found HPIV infections in all ages, with a more even distribution across age groups for HPIV2 in contrast to the more pronounced excess of HPIV4 in the under 9 and over 40 year old extremes of age, in general agreement with the epidemiological profile seen in the immediately preceding 15 year time period in the UK.^3^

HPIV4 is classically described as a widespread but mild, self‐limiting infection in contrast to HPIV2 with a strong etiological and epidemiological association with croup in infants (reviewed in^2^). Whilst we did see proportionately less hospitalisation with HPIV4‐infected individuals, overall severity was comparable to HPIV2 in our mono‐infected and hospitalised sub‐cohort with a similar need for intervention with nebulisers and oxygen, alongside shortness of breath. Similarly Frost et al.^9^ noted more hypoxia in HPIV4‐positive individuals compared to HPIV2 in children and generally similar severity between HPIV types. Fever was seen much more predominantly as a feature of HPIV2, but not HPIV4, infection, a trend previously noted by others, but without significance^9^, ^28^ perhaps due to cohort limitations. C‐reactive protein has previously been noted as collectively elevated by HPIV infection,^35^ and mildly elevated during HPIV4 and not HPIV2 infection in children. In contrast, we found a significant elevation collectively in children and adults in HPIV2 and not HPIV4 infection.

A few limitations were apparent in our investigations. Clinical details were not recorded for the purpose of this study and thus recorded in a non‐uniform and sometimes incomplete manner, reducing the availability of useable data and in turn statistical power. Even in these circumstances were able to achieve numbers of HPIV2 or HPIV4 mono‐infected individuals requiring hospital treatment equivalent to or exceeding the previous largest in‐depth cohorts published.^9^, ^16^, ^22^, ^28^ Collectively all these studies and ours fail to investigate the complete epidemiological burden of HPIV infections, with access only to clinical cases.^31^ Extended community surveillance of asymptomatic or sub‐clinical infection would greatly enhance knowledge of circulating HPIV strains, seasonal prevalence and associated pathogenesis, but will require considerable sampling effort.^36^ Potentially related to limited pathogenesis in the community is the paucity of reference material with which to inform primer design and potentially sample degradation in storage ‐ we, like others^16^ struggled to amplify certain portions of the HPIV genomes. Furthermore, many of the archetypal^13^ and also some more contemporary reference sequences^14^, ^15^, ^37^ have been generated from cell cultured viruses, which may cause genomic changes and potentially affect chosen PCR priming sites. The additional primers and sequences described in this study, particularly for HPIV4, provide further tools for future PCR‐based studies, whilst increasingly prevalent use of alternative deep sequencing strategies will further enhance knowledge of parainfluenza sequence diversity.^38^

## 5 CONCLUSION

In summary, we found HPIV2&4 in the East Midlands of the UK between 2013 and 2017 to be caused by multiple co‐circulating viral clades in adults and children. Both HPIV2&4 were frequently associated with hospitalised patients and occasionally severe disease. The general picture of respiratory disease in these individuals was distinguished by more frequent fever, abnormal haematology and elevated C‐reactive protein in HPIV2‐positive individuals.

With the recent disturbance to typical transmission of endemic human respiratory viral infections caused by non‐pharmaceutical intervention measures taken to control the SARS‐CoV‐2 pandemic, future orthorubulavirus epidemic patterns are uncertain and should be monitored carefully.^39^ However, the exceptional focus applied to the understanding and treatment of COVID‐19 could yield advances in the management of patients with HPIV2&4.^40^

## AUTHOR CONTRIBUTIONS

Akhil Chellapuri & Matthew Smitheman: Data curation & Investigation (Equal); Formal analysis, Writing – original draft, Writing – review & editing (Supporting). Joseph G. Chappell: Investigation, Methodology, Writing – review & editing (Supporting). Gemma Clark & Hannah C. Howson‐Wells: Resources (Equal), Data curation, Writing – review & editing (Supporting). Jonathan K. Ball: Conceptualization, Funding acquisition (Equal); Supervision & Writing – review & editing (Supporting). William L. Irving: Conceptualization, Resources, Supervision, Writing – review & editing (Supporting). Alexander W. Tarr: Formal analysis (Equal); Data curation, Supervision, Writing – review & editing (Supporting). C. Patrick McClure: Conceptualization, Data curation, Methodology, Project administration, Supervision, Visualization, Writing – original draft, Writing – review & editing (Lead); Formal analysis, Investigation (Equal).

## CONFLICT OF INTEREST DISCLOSURE

The authors have no relevant conflicts as outlined by the ICMJE to declare.

## ETHICS APPROVAL STATEMENT

Use of residual diagnostic nucleic acids and associated anonymized patient information was covered by ethical approval granted to the Nottingham Health Science Biobank Research Tissue Bank, by the North West ‐ Greater Manchester Central Research Ethics Committee, UK, reference 15/NW/0685.

## PATIENT CONSENT STATEMENT

Requirement for patient consent was waived under the above ethical approval reference 15/NW/0685.

## PERMISSION TO REPRODUCE MATERIAL FROM OTHER SOURCES

No material is reproduced from other sources.

## Supporting information


**Table S1:** PCR Primers utilised in the study.
**Table S2:** Ranges of normal values used for auditing of clinical features

## Data Availability

The genetic data that support the findings of this study are openly available in Genbank at https://www.ncbi.nlm.nih.gov/genbank/, accession numbers MZ576382:MZ576430. The additional data that support the findings of this study are available from the corresponding author upon reasonable request.
