# Supplementary material for: Human parainfluenza 2 & 4: Clinical and genetic epidemiology in the UK, 2013–2017, reveals distinct disease features and co‐circulating genomic subtypes
Source: Influenza Other Respir Viruses. 2022 Jun 7;16(6):1122–32. doi: 10.1111/irv.13012 (PMC9530586; doi:10.1111/irv.13012)
Supplement: Supplementary file 1 — Table S1: PCR Primers utilised in the study. Table S2: Ranges of normal values used for auditing of clinical features [file IRV-16-1122-s001.docx]

**Supplementary information**

**Supplementary methods**

**Supplementary Table 1:** PCR Primers utilised in the study.

| Primer Name | Sequence (5’ to 3’) | Reference genome coordinates | Annealing temperature (^o^ C) |
| --- | --- | --- | --- |
| AVU-RUB-F2^a^ | ACACTCTATGTIGGIGAICCNTTYAAY CC | 10925 - 10953 | 50 |
| AVU-RUB-R^a^ | GCAATTGCTTGATTITCICCYTGNAC | 11123 - 11148 | 50 |
| PF2_Fs | TCACCTGCATCCAATGATAGTAT | 4798 – 4820 | 61 |
| PF2_Fas | TGCATGTACATTGGGGAAATKGA | 5607 - 5585 | 61 |
| PF2_Fs2 | AGAATYCTCCTYGGTAGCAC | 5477 - 5496 | 61 |
| PF2_Fas2 | TGATAGAATTCTTAAGATATCCCATATATGTT | 6459 – 6428 | 61 |
| PF4_Fs | TGAATCTAGGAACGGTACCRAC | 5253 - 5274 | 61 |
| PF4_Fas | ACTGTATCTTTYGTGATTTGGCA | 6168 - 6146 | 61 |

Reference genome coordinates were determined for HPIV2 reference genome V94 (GenBank accession AF533010) and HPIV4 M-25 (AB543336). Nucleotides listed using standard IUPAC notation, with I denoting inosine bases. ^a^ Primers from Tong *et al* 2008 ^27^

**Supplementary Table 2:** Ranges of normal values used for auditing of clinical features

| **Haematology** | **Birth** | **2 weeks** | **4 weeks** | **2-6 months** | **6 months -1 Year** | **1-6 years** | **6-12 years** | **12-18 years** | **Adults** |
| --- | --- | --- | --- | --- | --- | --- | --- | --- | --- |
| Haemoglobin (g/L) | 140-240 | 134-198 | 134-198 | 94-130 | 111-141 | 115-140 | 115-155 | 120-160 (F)  130-170 (M) | 115-165 (F)  130-180 (M) |
| Platelets (x10^11^/L) | 1.5-4.0 | 1.5-4.0 | 1.5-4.0 | 1.5-4.0 | 1.5-4.0 | 1.5-4.0 | 1.5-4.0 | 1.5-4.0 | 1.4-4.0 |
| Red Blood Cells  (x10^12^/L) | 3.7-6.5 | 3.9-5.9 | 3.2-5.9 | 3.1-4.3 | 4.1-5.3 | 3.9-5.3 | 4.0-5.2 | 4.1-5.1 (F)  4.5-5.3 (M) | 3.8-5.8 (F)  4.5-6.5 (M) |
| White Blood Cells  (x10^9^/L) | 10.0-26.0 | 6.0-21.0 | 6.0-21.0 | 5.0-21.0 | 6.0-17.5 | 5.0-17.0 | 4.5-14.5 | 4.5-13.0 | 3.6-11.0 |
| Neutrophils (x 10^9^/L) | 2.7-14.4 | 1.5-5.4 | 1.5-5.4 | 1.0-5.0 | 1.0-8.5 | 1.0-8.5 | 1.0-8.0 | 1.5-8.0 | 1.8-7.5 |
| Lymphocytes (x10^9^/L) | 2.0-8.0 | 2.8-9.1 | 2.8-9.1 | 4.0-10.0 | 4.0-12.0 | 1.5-9.5 | 1.5-7.0 | 1.1-4.5 | 1.0-4.0 |
| Monocytes (10^9^/L) | 0-2.0 | 0.1-1.7 | 0.1-1.7 | 0.4-1.2 | 0.2-1.0 | 0.2-1.0 | 0.2-1.0 | 0.2-1.0 | 0.2-0.8 |
| Eosinophils (x 10^9^/L) | <0.81 | <0.91 | <0.91 | <0.81 | <0.81 | <0.81 | <1.01 | <0.81 | 0.1-0.4 |
| Basophils (x 10^9^/L) | <0.21 | <0.21 | <0.21 | <0.21 | <0.21 | <0.21 | <0.21 | <0.21 | 0.02-0.1 |
|  |  |  |  |  |  |  |  |  |  |
| **Liver and Kidney function** |  |  |  |  |  |  |  |  |  |
| AST (U/L) | <30 (F)  <35 (M) | <30 (F)  <35 (M) | <30 (F)  <35 (M) | <30 (F)  <35 (M) | <30 (F)  <35 (M) | <30 (F)  <35 (M) | <30 (F)  <35 (M) | <30 (F)  <35 (M) | <30 (F)  <35 (M) |
| ALT (U/L) | <35 (F)  <45 (M) | <35 (F)  <45 (M) | <35 (F)  <45 (M) | <35 (F)  <45 (M) | <35 (F)  <45 (M) | <35 (F)  <45 (M) | <35 (F)  <45 (M) | <35 (F)  <45 (M) | <35 (F)  <45 (M) |
| AST:ALT ratio | 0.8-1.0 | 0.8-1.0 | 0.8-1.0 | 0.8-1.0 | 0.8-1.0 | 0.8-1.0 | 0.8-1.0 | 0.8-1.0 | 0.8-1.0 |
| Albumin (g/L) | 35-52 | 35-52 | 35-52 | 35-52 | 35-52 | 35-52 | 35-52 | 35-52 | 35-52 |
| Bilirubin (µmol/L) | <21 | <21 | <21 | <21 | <21 | <21 | <21 | <21 | <21 |
| CRP (mg/L) | <10 | <10 | <10 | <10 | <10 | <10 | <10 | <10 | <10 |
|  |  |  |  |  |  |  |  |  |  |
| **Urea and Electrolytes** |  |  |  |  |  |  |  |  |  |
| Sodium (mmol/L) | 134-145 | 134-145 | 134-145 | 134-145 | 134-145 | 134-145 | 134-145 | 134-145 | 134-145 |
| Potassium (mmol/L) | 3.5-5.3 | 3.5-5.3 | 3.5-5.3 | 3.5-5.3 | 3.5-5.3 | 3.5-5.3 | 3.5-5.3 | 3.5-5.3 | 3.5-5.3 |
| Urea (mmol/L) | 0.4-4.3 | 0.4-4.3 | 0.4-4.3 | 2.0-5.3 | 2.0-5.3 | 2.0-5.3 | 2.0-5.3 | 2.0-5.3 | 2.0-6.5 (<60Yr)  2.9-7.5 (>60Yr) |
| Creatinine (µmol/L) | 27-81 | 14-34 | 14-34 | 14-34 | 14-34 | 15-42 | 30-64 | 38-81 (F)  38-99 (M) | 45-84 (F)  59-104 (M) |

*AST - aspartate aminotransferase,* ALT alanine aminotransferase, CRP C-reactive Protein
